# Outpatient Surgery in Neuro-Oncology—Advancing Patient Access and Care

**DOI:** 10.3390/curroncol33010040

**Published:** 2026-01-12

**Authors:** Patrick E. Steadman, Mark Bernstein

**Affiliations:** 1 Division of Neurosurgery, Temerty Faculty of Medicine, University of Toronto, Toronto, ON M5S 1A8, Canada; patrick.steadman@mail.utoronto.ca; 2 Division of Neurosurgery, Toronto Western Hospital, University Health Network, Toronto, ON M5G 2C4, Canada

**Keywords:** biopsy, brain tumor, complications, day surgery, Ommaya reservoir, outpatient, resection, surgery, resource utilization

## Abstract

Outpatient neurosurgical oncology has expanded with advances in anesthesia, imaging, and minimally invasive techniques, enabling safe same-day discharge for selected patients undergoing procedures such as stereotactic biopsy and craniotomy. In this review, we find that across multiple international series, same-day discharge rates in several studies ranging from 85 to 95%, with low complication (3–6%) and readmission rates when structured pathways, including standardized selection criteria, enhanced recovery protocols, and routine 4-h postoperative CT imaging, are used. Studies on economic analyses demonstrate substantial cost savings driven by reduced inpatient bed utilization, with no increase in adverse events. Key challenges identified include medicolegal concerns amongst physicians, patient education, and limitations in organization adoption. Telemedicine and remote monitoring are increasingly incorporated to streamline preoperative evaluation and postoperative follow-up, improving access and continuity of care. Emerging technologies such as laser interstitial thermal therapy and focused ultrasound may further expand the outpatient neuro-oncology repertoire. Overall, current evidence supports outpatient neurosurgical oncology as a safe, efficient, and patient-centered model when applied with structured clinical pathways and patient selection.

## Simple Summary

Advances in brain tumor care such as better imaging, safer anesthesia, and minimally invasive surgical tools now make it possible for some neurosurgery patients to go home the same day as their procedure. This review explains how carefully selected patients can safely recover at home after operations like craniotomy or brain biopsy, supported by close monitoring, clear instructions, and early follow-up. Research from many countries shows that outpatient neurosurgery can reduce costs, shorten hospital stays, and improve comfort without increasing complications. Some challenges remain to ensure surgeon confidence in this pathway, and appropriate patient education, the growing use of enhanced recovery pathways, and telemedicine are helping make outpatient neurosurgical oncology a safe, efficient, and patient-centered approach to care.

## 1. Introduction

The care of neuro-oncology patients has markedly progressed in the last 25 years with the advent of new medication therapies, surgical technologies, and radiotherapy paradigms [1]. This has occurred as oncological care has become increasingly collaborative with multiple disciplines coordinating to guide patient care. To ensure patient values, which often include optimization of family and home time, there are clear patient-centered benefits to ambulatory surgery.

Providing surgery in an outpatient or ambulatory setting allows patient recovery at home, eases access to quality-of-life benefits of family, personal space, and can be performed safely through patient selection, telemedicine, and surgical planning [2,3]. Furthermore, prior work has shown this confers a system cost benefit [4,5].

In this review we cover the progress made in outpatient neurosurgical interventions for oncology patients, including the current practices, benefits, and challenges. We highlight the growing array of tools that enable quicker access and recovery. This enables quality care with optimized patient quality of life.

## 2. Neurosurgery and the Advent of Outpatient Interventions

The idea of outpatient surgery is not new and is used regularly by many surgical specialties [2]. It increases access to procedures partly by reducing costs both to the facility and patient/insurer [4,5]. Most often outpatient surgery occurs in ambulatory surgical centers (ASC). In neurosurgery, these often include spine surgery, but others have been proposed given recent advances in neurosurgical technology [2]. Technological progress has enabled several neurosurgical subspecialties access to outpatient procedures. This includes functional neurosurgery with Radiofrequency Ablation, Laser Interstitial Thermal Therapy (LITT), Deep Brain Stimulation, Magnetic Resonance-guided Focused Ultrasound (MRgFUS), and Gamma Knife radiosurgery. Another area includes cerebrovascular neurosurgery with angiograms and endovascular treatment of some vascular pathologies [6].

Ambulatory centers have been proposed to cost 62–84% less compared to hospital based [7]. The most common ACSs for neurosurgery would be spine. These centers typically perform non-fusion procedures like laminectomy and discectomy, though efforts to expand include limited instrumented fusions. In these circumstances patients with fewer comorbities, lower ASA (American Society of Anesthesiologists) score, and younger age are often chosen [7]. The lesson from these examples is that patient selection, interdisciplinary communication, and close clinic or virtual follow-up leads to fewer conversions to inpatient care and equivocal patient outcomes.

Outpatient surgical care in neurosurgery has reliably found cost reduction of 30–40% [2]. The increase in ASCs has proposed the idea of including experiences as part of residency training. This would allow residents to identify patients and procedures suitable for outpatient setting and understand the practice model while increasing access [2]. This could lead to more surgical practices where delays and cancelations due to bed and/or staffing shortages are avoided by moving procedures out of the hospital into an ASC.

The role for ASC in oncology would first need standardized outpatient care. Thankfully, despite the inherent complexity of neurosurgical oncology, providing outpatient neurosurgical oncology care is not a new concept. Prior reviews and case series have trialed the practice [8,9]. Outpatient neuro-oncological surgery was pioneered in Toronto, Canada in 1996 [4,8,9,10,11,12,13,14,15,16,17,18]. Outpatient neurosurgical oncology has been implemented in both high-income countries such as Canada [4,8,9,10,11,12,13,14,15,16,17,18], the United States [5,19,20] and the United Kingdom [21], as well as resource constrained low-middle income countries like Nigeria [22] and India [23]. Despite these early examples, expansion of the practice has been limited.

The implementation often includes the use of a surgical day unit (SDU) with 2 h spent by the patient in the post-anesthetic care unit (PACU) followed by minimum of 4 h in the SDU. A post-operative CT performed 4 h post procedure, and if patients meet discharge criteria and CT was without issue, then the patient could be discharged [8]. In the United Kingdom cohort, 30 brain biopsies and 11 craniotomies were performed without adverse outcome [21]. From this group, 9 of the 11 craniotomies, and 27 of 30 brain biopsies were discharged 6 h post-operatively. In resource limited settings, the Nigeria implementation involved two patients and had no complications [22]. These represent early examples of outpatient oncology, but now 7–15 years later the field of neuro-oncology has advanced tremendously. The field now has improved surgical and anesthetic techniques, innovative technology, and enhanced collaborative care, the next sections examine the current practice landscape for outpatient neurosurgical oncology, the strengths, and challenges to growth while looking towards the future.

## 3. Current Practices in Outpatient Surgery in Neuro-Oncology

The most common implementations of outpatient surgery in neuro-oncology include craniotomy for tumor resection and stereotactic brain biopsy. Understanding the state of each, new emerging technologies will identify directions neurosurgical oncology may grow.

### 3.1. Craniotomy

The expansion of outpatient neurosurgery has increasingly encompassed craniotomies, supported by enhanced-recovery pathways, improved perioperative monitoring, and refined patient-selection strategies. These can include general anesthetic (GA) or awake procedures. Initial experience was cautiously adopted, but accumulating evidence now demonstrates that appropriately selected patients undergoing tumor resection or open biopsy can be safely discharged on the same day, with complication rates comparable to or lower than inpatient management.

Early feasibility was first reported by Bernstein in 2001 [10], who reported safe same-day discharge in a pilot cohort of 46 patients. Over subsequent decades, larger series validated these observations. One series of 136 awake craniotomies and 117 awake biopsies found that although transient neurological worsening occurred in approximately 5% of cases, there were no adverse events attributable to outpatient management [15]. A later paper analyzed 1003 cranial procedures including 249 craniotomies, achieving a 93% same-day discharge rate with only 2% unplanned readmissions; postoperative CT at four hours and standardized neurological assessments formed the backbone of their protocol [17]. Similarly, Au et al. evaluated 318 craniotomies, 141 under GA, and attempted same-day discharge in 44 cases with an 86% success rate [13]. Failures were driven primarily by postoperative seizures rather than protocol insufficiency. Complimenting these papers is a qualitative analysis that patients undergoing awake craniotomy value same-day discharge when patient education is provided. Close communication with the care team throughout and trust in the surgical process are factors that help patients focus on their disease rather than the logistics of the operation [24].

There is more recent data implementing formal Enhanced Recovery After Surgery (ERAS) pathways. One report described 175 of 198 (88.4%) patients selected for outpatient craniotomy were successfully discharged the same day with a low readmission rate of 2.9% [18]. Only six postoperative hemorrhages occurred, none attributed to premature discharge. Nijs et al. evaluated 630 same-day craniotomy candidates (491 awake, 139 GA), achieving a 91% success rate; failures were largely due to postoperative nausea/vomiting, transient neurological deficits, seizures, or social factors [11].

A combination of prospective and retrospective analyses published in the last 3 years have helped structure modern practice. Vallejo and colleagues found that among 334 surgical oncology patients, 37 met outpatient criteria and 32 (86%) were discharged as planned, with no complications [19]. Another study expanded this to 202 patients undergoing outpatient tumor surgery or endoscopic third ventriculostomy, achieving an 88% same-day discharge rate with a 1.5% complication rate [5]. A notable feature of their workflow was preoperative admission the night before for imaging and medical clearance. Their risk-stratification system identifies factors predictive of successful same-day discharge: extra-axial pathology, right-sided lesions, lack of prior treatment, and small preoperative tumor volume (<11.75 cm^3^). Higher Karnofsky Performance Status (KPS) scores also correlated strongly with success [25]. Similarly in a European context, craniotomies with ERAS and a ‘hospital-at-home’ model achieved 89% same-day discharge without complication [26].

**Figure 1:**
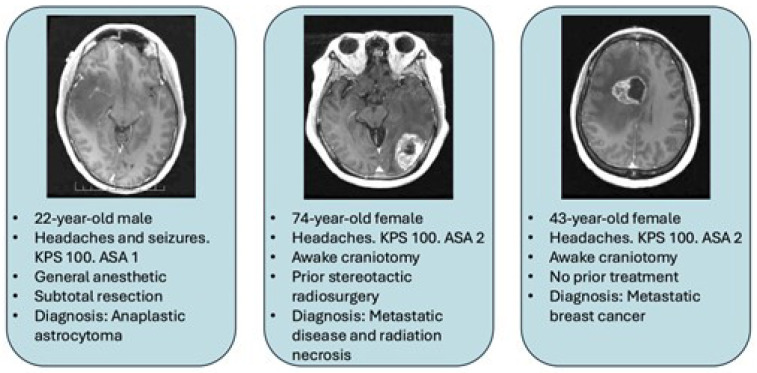
Illustrative cases where outpatient surgery was performed in neuro-oncology. A variety of tumor pathology, location, patient demographics, and anesthetic plan was used.

The implementations published examine a variety of pathologies. This includes intra- and extra-axial pathologies; some examples can be found in these references [5,11,17,18], and we provide three below in Figure 1. The only tumors which would necessarily exempt a patient from day surgery management are those where the anticipated post-operative course would require at least one night observation. This would, for example, include most skull base and posterior fossa tumors. For intra-axial tumors large size and edema are not necessarily reasons for inpatient care. Therefore, the key to proper safe implementation is patient selection and ERAS protocols which rely more on KPS, ASA, supratentorial lesion, and tumor size than discrete pathology [12,25]. The first case on the left side of Figure 1 shows T1 axial gadolinium-enhanced MRI of a 22-year-old man with new seizures and headaches. He underwent an aggressive subtotal resection of an anaplastic glioma which was performed under general anesthetic as an outpatient. The middle vignette shows a T1 axial gadolinium-enhanced MRI of a 74-year-old woman with headaches. She had received fractionated stereotactic radiosurgery (21 Gy in 3 fractions) 7 months prior to the left occipital breast cancer metastasis. The lesion had extensive edema. Resection of the lesion showed a mixture of recurrent tumor and radiation necrosis which was performed as an awake craniotomy as an outpatient. The rightmost vignette in Figure 1 shows a T1 axial gadolinium-enhanced MRI of a 43-year-old woman with breast cancer and new headaches. The lesion was resected with an awake craniotomy as an outpatient procedure.

#### 3.1.1. Benefits and Rationale

From an anesthetic and health systems perspective, outpatient craniotomy offers several advantages. Benefits include reduced cost, decreased risk of nosocomial infections (approaching 9–10% in some inpatient series), optimized resource utilization, and high patient satisfaction, when appropriate patient education and support provided [12]. Enhanced-recovery anesthesia, emphasizing normovolemia, rapid emergence, effective analgesia, and meticulous control of nausea, has facilitated reproducible same-day discharge without compromising safety.

#### 3.1.2. Patient Selection Criteria

Outpatient craniotomy under general anesthesia has become increasingly feasible due to refined perioperative pathways such as the Miami same-day discharge (SDD) score, multi-institution ERAS protocols, and multi-institutional ambulatory neurosurgery cohorts. Across these systems, appropriate candidates typically present with supratentorial lesions, most commonly extra-axial tumors or small intra-axial masses with low expected blood loss and operative times under three to four hours. Patients generally fall within a broad but functionally intact age range, carry ASA classifications IV or lower, and maintain a KPS ≥ 70 with stable neurological exam. Exclusions consistently include large tumor volume or significant mass effect, uncontrolled seizures, medical or neurocognitive instability, poor airway safety, or social limitations such as absence of overnight support or geographic remoteness [18,25,26]. Importantly, patient preference influences eligibility, as successful outpatient pathways depend on patient comfort with same-day discharge and reliable caregiver participation.

Postoperative management is a defining component of safe same-day discharge after craniotomy, with all published programs emphasizing structured, protocolized monitoring. Standard practice includes a minimum of six hours of observation in PACU and/or a step-down setting with frequent neurological checks, early postoperative CT imaging at approximately four hours, and strict control of pain, nausea, and blood pressure. Before discharge, patients must ambulate, tolerate oral intake, and void independently. Comprehensive discharge instructions including clear red-flag symptoms and 24/7 contact pathways are universal requirements. Follow-up typically involves a next-day phone call by the surgical or ambulatory nursing team, early postoperative clinic review within 24–72 h, and in some centers, short-term hospital-at-home nursing visits for additional monitoring. Together, these measures create a reliable safety net that enables outpatient general-anesthetic craniotomy to be performed safely in appropriately selected patients.

Across diverse centers and practice models, outpatient craniotomy under general anesthesia has evolved into a safe, efficient option for well-selected patients with brain tumors. Standardized pathways anchored in rigorous selection, focused anesthetic management, and structured postoperative assessment allow same-day discharge rates of 85–95% with low readmission and complication rates. As patient expectations and healthcare resource pressures continue to evolve, outpatient craniotomy is poised to become an integral component of modern neurosurgical oncology care.

### 3.2. Brain Biopsy

Stereotactic brain biopsy is a cornerstone diagnostic procedure in neuro-oncology, enabling tissue acquisition for histopathological and molecular characterization when definitive resection is not appropriate. Because the goal is diagnostic clarity rather than cytoreduction, and because the procedure is minimally invasive with a predictable postoperative course, it has emerged as an attractive target for outpatient neurosurgery programs. Over the past three decades, multiple centers have evaluated the safety, feasibility, and workflow requirements of same-day discharge after brain biopsy, collectively building a substantial evidence base supporting outpatient management.

Early investigations established both feasibility and acceptable complication rates. One of the first studies reported an outpatient rate of 81% among 71 patients at the Cleveland Clinic, and a 6% symptomatic complication rate, including one permanent deficit, two transient hemorrhage-related deficits, and one cerebral abscess [27]. Of note is that many of these patients were discharged before 24 h; in other words, they did spend a night in hospital and were not true outpatients. The first truly outpatient biopsy group was reported from Toronto. There 76 patients were analyzed in the intent-to-treat outpatient biopsy group; same-day discharge was achieved in 97% of those cases, with only a 3% symptomatic complication rate [14]. This study demonstrated that carefully managed outpatient pathways could achieve safety outcomes comparable to inpatient observation.

Subsequent work confirmed these findings across health systems. A United Kingdom study found a 45% outpatient rate among 30 patients with a 90% successful discharge rate and a single symptomatic seizure [21]. In a large series of over 1000 patients which included 152 biopsies, reporting a 62% outpatient rate, 94% successful discharge, and a 6% symptomatic complication rate [17]. A recent European study focused on highly selective ambulatory pathways. These data applied stringent selection criteria to a prospective cohort of 40 patients, resulting in a low 9% outpatient rate [28]. Notably, all outpatient cases achieved successful same-day discharge, and no symptomatic complications were observed.

Taken together, the international literature demonstrates that outpatient stereotactic brain biopsy is feasible and safe, with symptomatic complication rates between 3% and 6% and very low mortality. Differences in same-day discharge rates across centers are attributable primarily to institutional infrastructure, selection criteria, and cultural adoption rather than procedural risk. Nonetheless, it is clear there are patients suitable for outpatient biopsy regardless of institutional variances.

### 3.3. Gamma Knife Radiosurgery, MRgFUS and LITT

Gamma Knife radiosurgery (GKRS) has long been established as a predominantly outpatient neurosurgical intervention, owing to its minimally invasive, non-incisional nature and predictable peri-procedural course. Most patients are discharged the same day of frame placement, stereotactic imaging, treatment planning, and radiosurgical delivery. Several studies have trialed same-day GKRS treatment following stereotactic cyst aspiration for cystic brain tumors [29]. The study did not explicitly document same-day discharge, though overwhelmingly GKRS is ambulatory. The reliability of procedural workflow, low complication rates, and absence of general anesthesia support its integration into outpatient neurosurgical oncology, where it is used for brain metastases, meningiomas, vestibular schwannomas, and highly selected gliomas.

Laser interstitial thermal therapy (LITT) represents another minimally invasive technique with expanding applications in neuro-oncology, including deep-seated gliomas, recurrent metastases, and radiation necrosis. Historically, LITT has required brief postoperative hospitalization, largely due to the need for observation following general anesthesia and monitoring for delayed edema. Studies have reported postoperative stays of one to two days in patients undergoing biopsy followed by LITT, with 85% discharged within 48 h and a complication rate of 4% [30]. However, emerging data suggest true outpatient LITT is possible. A 2025 study of a same-day discharge protocol in 10 patients demonstrated no differences in postoperative KPS or complication rates compared to a matched cohort discharged on postoperative day one [20].

Additionally, other new neurosurgical tools such as MR-guided focused ultrasound blood allow brain barrier opening for chemotherapy delivery [31,32] and for biopsy using circulating DNA [33]. Although still experimental, this approach is conceptually well-suited to ambulatory workflows. Collectively, these advances illustrate a broader trend: as technologies become more precise and perioperative pathways more structured, many neuro-oncologic interventions are shifting toward safe same-day treatment models.

### 3.4. Pediatrics

Outpatient neuro-oncology surgery in pediatric patients is special due to clinical complexity and postoperative monitoring needs of the pediatric population. Very early work demonstrated promising feasibility with 31 children having outpatient pediatric neurosurgery; there were no anesthetic complications and only one unplanned admission [34]. Furthermore, the study found substantial cost savings when procedures were performed in an outpatient facility. However, little subsequent research has focused specifically on pediatric oncologic indications.

In non-oncologic areas, such as minimally invasive craniosynostosis surgery, same-day discharge has become increasingly common [35]. Nevertheless, pediatric oncology patients often present with higher medical acuity, challenges in neurological assessment, and great parental concern, making broad adoption of outpatient neurosurgical pathways less likely. As a result, ambulatory neurosurgery in children will likely remain limited to carefully selected low-risk procedures.

## 4. Benefits of Outpatient Surgery in Neuro-Oncology

The shift toward outpatient surgery in neuro-oncology offers several compelling advantages for patients and healthcare systems. Same-day discharge models can promote faster recovery by minimizing hospital-associated deconditioning and reducing exposure to nosocomial risks, thromboembolic complications, medication errors, and other hospital-based morbidities. From a systems perspective, outpatient pathways allow more efficient use of constrained resources, particularly in settings where hospital beds and specialized nursing staff are limited. These efficiencies translate into meaningful cost reductions for both institutions and patients, making ambulatory neurosurgical oncology an increasingly attractive model of care. This might be particularly useful in low and middle-income countries where patients’ access to care also poses financial barriers and costs are less with outpatient surgery.

Economic analyses reinforce these advantages. A Canadian cost comparison demonstrated nearly a two-fold increase in expenses when brain tumor patients were admitted as surgical day admits rather than managed in an outpatient pathway, with no significant differences in complications or readmissions between groups [4]. The primary drivers of cost savings were reduced use of allied health services and avoidance of inpatient bed occupancy. Similar findings have been reported in broader neurosurgical cohorts, where outpatient management yielded notable reductions in cost without an associated rise in adverse events [2]. Together, these studies support the economic feasibility and safety of outpatient approaches in appropriately selected neurosurgical oncology patients.

Concerns about post-discharge complications and the need for unplanned admission remain central considerations in outpatient surgery. However, available data suggest these risks are low when clear selection criteria and postoperative pathways are in place. A 2016 study of 329 outpatient neurosurgical patients reported an unplanned admission rate of 2.9%, underscoring the safety of this model in well-chosen candidates [18]. Importantly, most published studies were conducted in hospital-based outpatient programs, where same-day admission is readily available if needed. A more recent analysis of 630 patients scheduled for same-day discharge found that only 9% ultimately required overnight observation, and all were discharged the following day [11]. Reasons for these short-stay admissions included transient neurological deficits, postoperative nausea and vomiting, seizures, and social factors such as patient/family preference. This suggests that many admissions reflect caution rather than major complications.

Overall, these findings highlight the potential for outpatient neurosurgical oncology to deliver high-quality, cost-effective care with low unplanned admission rates. As surgical techniques, anesthesia protocols, and perioperative pathways continue to advance, the benefits of outpatient neurosurgical oncology are likely to expand, offering a sustainable and patient-centered model for modern neuro-oncologic care.

## 5. Challenges of Outpatient Surgery in Neuro-Oncology

Despite growing evidence supporting the safety and efficiency of outpatient neurosurgical oncology, significant challenges continue to limit widespread adoption. Administrative and logistical barriers remain substantial. Successful outpatient programs require coordinated anesthesia protocols, well-equipped facilities with the ability to manage short postoperative observation periods, and robust pathways for patient education and buy-in. Many centers lack dedicated infrastructure or staffing to support same-day discharge workflows, and facility regulations in some jurisdictions restrict the duration of postoperative monitoring allowed in ambulatory surgery centers, thereby limiting which neurosurgical procedures can be legally or safely performed [36]. Patient hesitancy is another recurring barrier; in a European pilot program for outpatient craniotomy for supratentorial tumors < 3 cm, reluctance from both patients and clinical teams was cited as a major obstacle despite safe outcomes in the nine cases performed [37]. These concerns echo long-standing perceptions within neurosurgery. For example, one study found that although most surgeons agreed outpatient neurosurgery was feasible, only 6% routinely offered it, largely due to medicolegal anxiety and fears of rare but catastrophic delayed neurological deterioration, despite proven published track records of safety [38].

Technical and clinical considerations further complicate implementation. Patient selection remains critical: factors such as age, functional status, pre-existing hypertension, and ASA grade have been identified as predictors of safe same-day discharge [39]. Standardized postoperative imaging, such as a CT scan at approximately four hours, is widely accepted and supported by earlier evidence showing that most early complications occur within this window [3,8,16]. Telemedicine may help overcome some of the challenges inherent to frequent pre- and postoperative assessment. Virtual visits allow imaging review, neurological examination, and streamlined patient counseling, improving convenience and patient-centeredness [40]. Early postoperative follow-up, such as a structured phone call on postoperative day one, has been associated with higher patient satisfaction [41] and reduced readmission rates in other surgical populations [29]. Nevertheless, even with supportive technologies and standardized pathways, not all patients are appropriate candidates for outpatient surgery, and careful triage remains essential.

Overall, the obstacles to outpatient surgery in neuro-oncology are multifactorial, including administrative, technical, cultural, and medicolegal domains. Overcoming these challenges will require clear institutional pathways, expanded experience with outpatient workflows, patient education strategies that build confidence in same-day care, and adoption of supportive tools such as telemedicine. Continued accumulation of safety data and refinement of selection criteria will be key to shifting practice patterns and enabling broader implementation of outpatient neurosurgical oncology.

## 6. Considerations in Implementing an Outpatient Care Pathway

**Figure 2:**
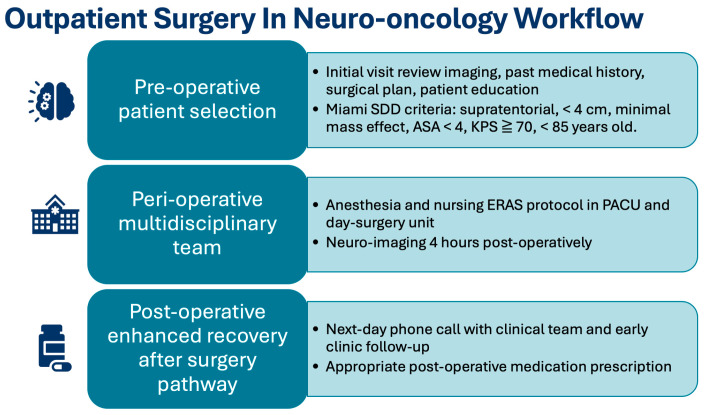
Summary of patient workflow and key features for an outpatient neuro-oncology surgical pathway.

Institutions that successfully implement outpatient surgical programs tend to share several structural and organizational features (Figure 2). First, a dedicated day-surgery unit experienced in perioperative neurosurgical care is essential. Second, the ability to schedule biopsies and craniotomies with sufficient time for post-operative monitoring before discharge is necessary. Third, strong coordination among neurosurgeons, anesthesiologists, and day-surgery nurses ensures consistent adherence to protocol. Fourth, the presence of a standardized, stepwise pathway, including operative workflow, postoperative neurological exams, and discharge criteria is critical [42]. Finally, a postoperative day-one telephone appointment helps to identify complications early and reinforces continuity of care.

### 6.1. Appropriate Patient Selection 

Proper patient selection is central to minimizing risk outside the hospital setting. Inclusion criteria commonly adopted across published programs include nearby residence of the treating center and the availability of a reliable adult caregiver for assistance in the first 24 h. These conditions ensure timely access to emergency care in the unlikely event of delayed hemorrhage or neurological deterioration.

Several exclusion criteria have been consistently applied—patient reluctance toward outpatient management, medical comorbidities requiring extended postoperative monitoring (particularly bleeding disorders), pre-existing inpatient status, baseline neurological impairment with a modified Rankin Scale score > 3, uncontrolled epilepsy, ASA score > 3, and advanced age (decided on an individual bases). Such criteria collectively reduce the likelihood of late postoperative complications that could require urgent intervention.

### 6.2. Patient Education and Preoperative Counseling

A defining feature of outpatient stereotactic biopsy programs is the emphasis on comprehensive patient and caregiver education. Preoperative counseling must include a clear description of the biopsy procedure, expected operative duration, and anticipated postoperative course. This should include explicit communication regarding the outpatient process, including same-day discharge expectations and home monitoring requirements.

Patients are educated about potential complications, such as seizures, delayed neurological deficits, and changes in consciousness, and are instructed on early warning signs that should prompt immediate medical evaluation. Programs also routinely provide essential contact information, including numbers for the neurosurgical team, and the 24/7 on-call neurosurgical resident. This structured educational approach reinforces patient trust and supports safe recovery at home.

The cumulative international literature supports outpatient stereotactic brain biopsy and craniotomy for select cases as a safe, efficient, and patient-centered alternative to routine inpatient observation. When delivered within institutions equipped with dedicated day-surgery infrastructure, standardized protocols, vigilant postoperative monitoring, and rigorous patient education, outpatient neurosurgery achieves low complication rates and high patient satisfaction. The major features of established care pathways from two major groups [12,25] are summarized in Table 1. Published series across North America and Europe demonstrate that symptomatic complication rates are comparable to inpatient management, and successful discharge rates consistently exceed 90% in mature programs.

Future developments in outpatient surgery in neuro-oncology will likely focus on standardizing clinical pathways across institutions, refining imaging-based same-day safety assessments, and integrating telemedicine-based postoperative neurological monitoring. Broader eligibility criteria may emerge as more centers gain experience and confidence, potentially expanding outpatient access to older patients or those with stable comorbidities. Additionally, future research should evaluate cost-effectiveness, caregiver burden, and long-term patient-reported outcomes to further validate outpatient biopsy as a value-based standard of care.

In summary, outpatient brain biopsy and craniotomy represents a natural next step in comprehensive neuro-oncology, offering high-quality care with reduced hospitalization, improved patient autonomy, and optimized resource utilization. As protocols mature and evidence continues to accumulate, outpatient pathways are poised to become a core component of modern neurosurgical oncology practice.

## 7. Conclusions and Opportunities in Outpatient Surgery in Neuro-Oncology

As neuro-oncology continues to evolve, timely assessment and treatment remain foundational to optimizing patient outcomes. When rigorous patient selection, coordinated perioperative care, and clear patient education are achieved, a pathway to deliver safe, efficient, and patient-centered treatment in an outpatient setting is possible. Across a diverse body of retrospective and prospective studies, complication rates for outpatient brain biopsy, craniotomy, and selected minimally invasive procedures remain low when standardized inclusion criteria and structured postoperative monitoring are applied. Cost analyses consistently demonstrate substantial savings, largely through reduced hospital bed occupancy and allied health utilization, without compromising safety or patient satisfaction. Nonetheless, several implementation challenges persist, including variation in institutional comfort, inconsistent patient education practices, and uncertainty regarding universal selection and discharge criteria. Summarized here from several recent papers are valuable frameworks for institutions seeking to build or refine outpatient neurosurgical oncology pathways, but broader validation remains necessary [25].

Looking ahead, the outpatient landscape is poised to expand beyond traditional procedures such as biopsy and craniotomy. Technologies including Laser Interstitial Thermal Therapy (LITT) and focused ultrasound hold promise for broadening the portfolio of outpatient neuro-oncologic interventions. Further prospective studies are required to define their perioperative safety profiles, refine workflow requirements, and identify which patients will benefit most from same-day care.

Future progress will also depend on embracing supportive technologies and redesigned care pathways. Telemedicine has demonstrated value in preoperative evaluation, postoperative surveillance, and enhancing patient-centered convenience. Engaging patients, allied health, nurses, other care providers, and hospital administrators is necessary to design appropriate care pathways [12,43]. Multi-center prospective studies can validate safety across diverse practice environments and harmonize existing protocols into reproducible, evidence-based pathways. As neurosurgical oncology integrates emerging technologies and remote care infrastructure, outpatient surgery stands to play an increasingly central role in delivering safe, cost-effective, and patient-centered cancer care.

## Figures and Tables

**Table 1:** Care pathways and patient selection criteria implemented in Toronto and Miami.

Toronto Same-Day Discharge Craniotomy Pathway [12]	Miami Same-Day Discharge (SDD) Selection Criteria [25]
**Pre-operative phase:** Selection: supratentorial, caregiver available at home, staying within 1 h drive, surgical duration < 4 h, no significant comorbidities or uncontrolled seizures or poor neurological function Patient preparation: pre-operative patient education with anesthesia and surgery.	**Inclusion:** Supratentorial lesion Patient age 16–85 years ASA class ≤ 4
**Intra-operative phase:** Anesthesia: awake or GA plan, invasive monitoring only if indicated, normovolemia, careful early emergence and extubation Surgery: minimal lesion targeted surgical flap, avoid hyperosmolar therapy, efficient closure, minimal blood loss	**Exclusion:** Maximal tumor diameter > 4 cm EBL > 300 mL Estimated OR time > 3 h
**Post-operative phase:** PACU: pain and blood pressure control (<160/90). Minimum 2 h observation DSU: CT at 4 h, neurological assessment, clear patient instructions, appropriate prescriptions, 24 h follow-up call and clinic appointment.	**Clinical score for SDD (range −2 to 2):** Left-sided lesion: − 1 Prior treatment: −1 Extra-axial lesion: +1 Tumor volume < 11.75 cm^3^: +1

## Data Availability

No new data were created or analyzed in this study. Illustrative cases are original and further information can be sent to the corresponding author.

## Abbreviations

The following abbreviations are used in this manuscript:

ASC	Ambulatory surgical center
LITT	Laser Insteritial Thermal Therapy
MRgFUS	Magnetic Resonance-guided Focused Ultrasound
ASA	American Society of Anesthesiologists
SDU	Surgical Day Unit
PACU	Post-anesthetic Care Unit
GA	General Anesthesia
ERAS	Enhanced Recovery After Surgery
KPS	Karnofsky Performance Status
SDD	Same-day discharge
CT	Computed Tomography
GKRS	Gamma Knife Radiosurgery
